# HP1β Is a Biomarker for Breast Cancer Prognosis and PARP Inhibitor Therapy

**DOI:** 10.1371/journal.pone.0121207

**Published:** 2015-03-13

**Authors:** Young-Ho Lee, Xiyong Liu, Fuming Qiu, Timothy R. O’Connor, Yun Yen, David K. Ann

**Affiliations:** 1 Department of Molecular Pharmacology, Beckman Research Institute, City of Hope, Duarte, California, United States of America; 2 Department of Medical Oncology, Second Affiliated Hospital, Zhejiang University, School of Medicine, Hangzhou, China; 3 Department of Cancer Biology, Beckman Research Institute, City of Hope, Duarte, California, United States of America; School of Medicine and Health Sciences, University of North Dakota, UNITED STATES

## Abstract

Members of the heterochromatin protein 1 family (HP1α, β and γ) are mostly associated with heterochromatin and play important roles in gene regulation and DNA damage response. Altered expression of individual HP1 subtype has profound impacts on cell proliferation and tumorigenesis. We analyzed the expression profile of HP1 family by data mining using a published microarray data set coupled with retrospective immunohistochemistry analyses of archived breast cancer biospecimens. We found that the patient group overexpressing *HP1β* mRNA is associated with poorly differentiated breast tumors and with a significantly lower survival rate. Immunohistochemical staining against HP1α, HP1β and HP1γ shows that respective HP1 expression level is frequently altered in breast cancers. 57.4 - 60.1% of samples examined showed high HP1β expression and 39.9 - 42.6 % of examined tumors showed no or low expression of each HP1 subtype. Interestingly, comparative analysis on HP1 expression profile and breast cancer markers revealed a positive correlation between the respective expression level of all three HP1 subtypes and Ki-67, a cell proliferation and well-known breast cancer marker. To explore the effect of individual HP1 on PARP inhibitor therapy for breast cancer, MCF7 breast cancer cells and individually HP1-depleted MCF7 cells were treated with PARP inhibitor ABT-888 with or without carboplatin. Notably, HP1β-knockdown cells are hypersensitive to the PARP inhibitor ABT-888 alone and its combination with carboplatin. In summary, while increased HP1β expression is associated with the poor prognosis in breast cancer, compromised HP1β abundance may serve as a useful predictive marker for chemotherapy, including PARP inhibitors against breast cancer.

## Introduction

Breast cancer is one of the leading causes of death in the United States and worldwide. Early diagnosis and effective use of adjuvant therapies are required to improve patient survival [1, 2]. Prognostic factors that are frequently used for making clinical decisions in breast cancer are age, tumor size, status of lymph nodes, histological types of the tumor, pathological grade, and hormone receptor status. However, more biomarkers are needed for therapy and prediction of outcome because human breast cancers are diverse in their genetic nature and their response to therapy. Recently, many groups have tried to identify gene signatures of breast cancer patients [3, 4]. These gene signatures can lead to more accurate clinical decisions for cancer patients [5]. Breast cancer can be classified into several groups depending on their expressions of biomarkers and pathology of breast cancer specimens. The most common molecular markers for breast cancers include estrogen receptor (ER), progesterone receptor (PR), HER2/neu, EGFR, Ki-67 and others [6]. The subgroups of breast cancer include Luminal A, Luminal B, Basal, HER2-enriched subtypes [6]. Triple negative breast cancer subtypes, which have deficient expression of ER, PR and HER2/neu, usually have poor prognosis and do not respond to hormone therapy. However, triple negative breast cancer is also a heterogeneous group, which shows different gene signatures [7]. For example, some triple negative breast cancers have defective *BRCA1* genes, whereas other triple negative breast cancer patient groups have functional *BRCA1*. *BRCA1* is one of the most frequently mutated genes in breast cancer patients [8]. Women with germline mutations in *BRCA1* have high risk of breast cancer (~80% by the age of 70), ovarian cancer (~30–40%) and other cancers. BRCA1 is involved in maintaining genomic integrity by functioning in pathways involved in DNA repair, cell cycle checkpoint control, apoptosis, chromosome segregation and others [8]. One of the main roles of BRCA1 is to promote homologous recombination repair and G2/M cell cycle arrest during DNA damage response. Thus, the loss of BRCA1 is frequently associated with a dramatic increase of genomic instability and tumorigenesis. While germline BRCA1 mutations are rarely found in patients with sporadic breast cancers, the functions of BRCA1 may be inactivated by other mechanisms, which are often referred to as “BRCAness” [9]. One of the possible mechanisms of BRCAness is the inactivation of BRCA1 function at the epigenetic level by DNA methylation of the *BRCA1* promoter [9, 10].

BRCA status is also important for cancer therapy. The genomic instability of BRCA1- and BRCA2-defective cells can be exploited for cancer therapy [11, 12]. Clinically, the genomic instability phenotype of BRCA1- and BRCA2- deficient cells provided an opportunity for PARP inhibitor treatment [12, 13]. Poly(ADP-ribose) polymerase (PARP) is involved in the repair of DNA single strand breaks (SSBs), and failure of their repair can lead to the generation of DNA double strand breaks (DSBs) during DNA replication. Inhibition of PARP1 leads to a large increase in DSBs and to cell death in the absence of BRCA1 or 2 and/or in the absence of HR dependent DSB repair [11, 12]. This is the basis for the concept that PARP inhibitors induce synthetic lethality in HR repair deficient tumors and provides a novel strategy for cancer therapy, at least in breast cancer patients who have mutations in BRCA1 or BRCA2. Recent clinical trials of a PARP inhibitor reported a partial success in cancer therapy with less severe side effects [14–16].

Previously, we found that HP1 is an important factor for the activity of BRCA1 as part of the DNA damage response pathway [17]. In this study, we investigated the expression level of Heterochromatin protein 1 (HP1) in breast cancer cases. HP1 binds to dimethylated and trimethylated histone H3 (H3K9Me2 and Me3) and associates with heterochromatin in the nucleus [18, 19]. HP1 has diverse roles that include gene regulation and DNA damage response among others [20, 21]. We have recently shown that BRCA1 is not functional in its foci formation, homologous recombination repair, or G2/M checkpoint control in the absence of HP1 expression upon DSB induction [17]. Since HP1 is an essential factor for BRCA1 function during the DNA damage response pathway, it is possible that HP1 expression levels may be altered during tumorigenesis. Here, we found the heterogeneous expression of all three HP1 subtypes in breast cancer patients. We uncovered that breast cancer patients with tumors expressing high levels of *HP1β* mRNA had less probability of survival. We also found the positive correlation of HP1 expression and Ki-67 cancer marker in breast cancer samples, suggesting potential significance of HP1 as a marker for breast cancer prognosis. Furthermore, we showed that PARP inhibitor ABT-888 was more effective in inducing death of HP1β-deficient MCF7 breast cancer cells. These data suggest that HP1β level could not only serve as a useful marker for breast cancer prognosis but also as a predictive marker for PARP therapy.

## Materials and Methods

### Data mining on microarray dataset

A total of 10 published microarray data sets including: Ivshina (GSE4922), Chin (E-TABM-158), Wang (GSE2034), Pawitan (GSE1456), Desmedt (GSE7390), Expo (GSE2109), Huang [22], Bild (GSE3143), Sortiriou (GSE2990) and NKI [23] with clinical annotations were downloaded from the combined microarray dataset BRAVO (Biomarker recognition and validation on-line). The NKI (Netherlands Cancer Institute)-295 set was especially selected for HP1 prognostic evaluation because the probe (Agilent Technologies) for *cbx1* (HP1β) is 100% identical to previously identified sequence of *cbx1* and it contains information of most gene signatures’ classification. NKI data set (295 patients analyzed, Accession number N/A) used 25,000-gene array that comes from Agelent Technologies, which used same probers with Affymetrix HG-U133 array.

### Patient enrollment, follow-up and tissue array

Patients diagnosed with breast cancer and treated by surgical resection between January 2002 and January 2006 in the Second Affiliated Hospital of Zhejiang University (ZJU) were included in this study. A breast cancer pathologist (F. Q.) used haematoxylin and eosin (H&E)-stained slides, to retrospectively review the history of all cases. The clinicopathological parameters that were evaluated included patient age at the time of diagnosis, tumor node metastasis (TNM) stage, date of last follow-up, and overall patient survival. Exclusion included breast cancer samples from patients without a pathologic diagnosis, those with multiple cancers, or those patients with whom contact was lost after surgery. A total of 222 breast cancer patients were included in this study. Follow-ups were conducted for all participants and the surgery relapse and death data were collected until 2010. Overall survival (OS) rate was calculated from the date of surgery to date of death by breast cancer-associated illness. Disease-free survival (DFS) rate was calculated from date of surgery to date of local recurrence or metastasis. If no death or relapse occurred, the OS and DFS rates were calculated from date of surgery to September 2010. All of the formalin-fixed, paraffin-embedded (FFPE) breast cancer tissue samples that were collected were reassembled into multiple tissue arrays. Analysis indicated that HP1 immunohistological signals did not correlate with storage time (likelihood, *p* = 0.246), indicating the storage time did not affect the immunohistochemical outcome.

### Immunohistochemistry (IHC)

HP1 protein levels in the 222 breast cancer samples were assessed by IHC with anti-HP1 antibodies (1:75 dilution); anti-HP1α (Bethyl, Abcam), anti HP1β (ab10478, Abcam) and anti-HP1γ (ab10480, Abcam). The IHC conditions for HP1 expression determination were pre-optimized on checkboards with multiple tissue samples. Briefly, after de-paraffinization, pre-treatment with 3% H_2_O_2_ was used to block the endogenous peroxidase activity. The slides were incubated with normal goat serum for 20 minutes at room temperature (RT) to block non-specific signal, then incubated with the primary antibody for 20 minutes at RT. The array slides were then incubated with polymer horseradish peroxidase-labeled secondary antibodies for 30 minutes at RT, then 3,3-Diaminobenzidine (DAB)-treated (0.05 g DAB and 100 ml 30% H_2_O_2_ in 100 ml PBS) for 5 and 10 minutes, respectively. Each slide was counterstained with DAKO's haematoxylin. For each IHC staining, the negative and positive checkboards were applied as quality controls. The specificity of anti-HP1 antibodies were validated by Western analyses. HP1 staining was predominantly nucleus, and HP1 expression was assessed using a visual grading system on the basis of the intensity of staining signals observed by light microscopy. Each sample was independently scored by two investigators (Y.L. and X.L.), including one breast cancer pathologist (X.L.) using a double-blind design to avoid scoring bias. Discrepancies were re-evaluated by joint review between the two readers. Less than 10% variation was noticed among different slides.

### Statistical analysis

The database was created by using MS-Access and data analysis was performed using JMP 8.0 software (SAS Institution) and GraphPad Prism 5.0 software. Group comparisons for continuous data were done by t-test for independent means or 1-way ANOVA. Each cell biology experiment was performed in triplicate to obtain representative means and images. Categorical variables were compared using χ^2^ analysis, Fisher’s exact test or binomial test of proportions. Kaplan-Meier analysis and a COX hazard proportional model were used to analyze overall survival and disease-free-survival. Multivariate analysis and stratification were used to reduce the confounder’s impact on the estimation of the Hazard Ratio (HR). Statistical significant was set as *p* < 0.05, two-tailed.

### Apoptosis assay

Apoptotic cells were measured by FITC Annexin V Apoptosis Detection Kit I (BD Pharmingen) according to manufacturer’s protocol. MCF7 cells and HP1-depleted MCF7 cells were cultured and harvested before or after irradiation. The harvested cells were washed twice with ice-cold PBS and then resuspended cells in 1 x Binding Buffer (0.1 M Hepes/NaOH (pH 7.4), 1.4 M NaCl, 25 mM CaCl_2_.) at a concentration of 1 x 10^6^ cells/ml. 100 μl of cells (1 x 10^5^ cells) were transferred to a 5-ml culture tube and incubated with FITC-conjugated Annexin V (5 μl). The incubated cells were incubated for 15 minutes at Room Temperature (25°C) in the dark and 1 x Binding Buffer (400 μl) was added to each tube. The stained cells were analyzed by flow cytometry.

### Ethical statement

The protocol for the use of human tissues was reviewed and approved by the Medical Ethics committee of the 2nd affiliated hospital, school of medicine, Zhejiang University (Zhejiang, China) (Institutional Review Board (IRB) number 73, Approving date; Dec 12, 2012). Prior to the study, all patients gave their written informed consent to allow us to use leftover tissue samples for scientific research. All eligible participants had received modified radical mastectomy and the primary tumor samples were obtained from surgical specimens. The exclusion criteria were: 1) no informed consent obtained, 2) multiple cancers, 3) lack of histological diagnosis, and 4) no follow-up information.

## Results

### *HP1β/CBX1* mRNA level is inversely associated with breast cancer patient survival

**Fig 1:**
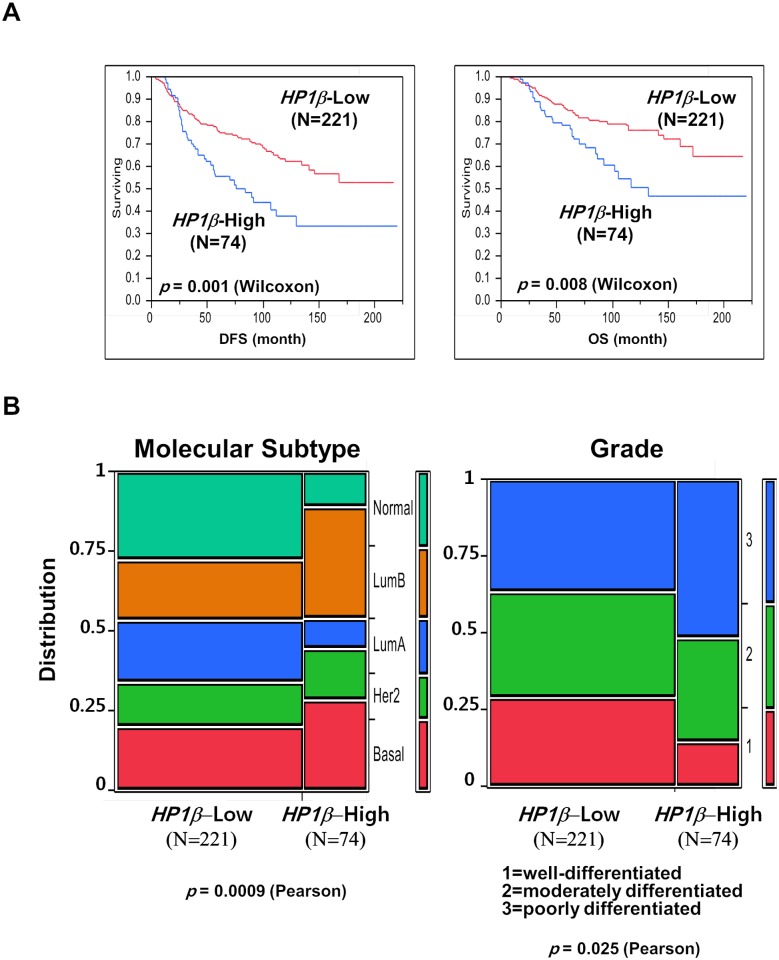
*HP1β* message abundance is associated with survival of breast cancer patients. A microarray database of 295 breast cancer patients (NKI-295 dataset) was analyzed and *HP1β* message signals were investigated. *A*. Kaplan-Meier analyses indicated that *HP1β* mRNA abundance is inversely correlated with both disease-free survival (DFS) and overall survival (OS) for breast cancer patients. *B*. Group of high *HP1β* expressors is associated with aggressive and poorly differentiated breast cancers. Low or high *HP1β* message abundance are denoted from microarray database from the public domain [23].

Initially, we used data mining techniques to determine if the expression level of HP1β/CBX1 mRNA was associated with the outcome of breast cancer patients using a published microarray dataset [23, 24]. Since the expression level of *HP1β* mRNA is diverse in breast cancer samples, we classified patients into four groups (0, 1, 2, 3) according to quartile of *HP1β* mRNA levels. A total of 74 breast cancer patients were stratified as high expressors of *HP1β* mRNA (group-3) and 221 patients were classified as low or no expressors of *HP1β* (group-0, -1 or -2). Kaplan-Meier analyses indicated that *HP1* expression was a critical prognostic indicator for both overall and disease-free survival for all breast cancer patients. Notably, high *HP1β* expressor group (N = 74) was associated with lower DFS (disease-free survival) (*p* = 0.001) and OS (overall survival) (*p* = 0.008), when compared with lower *HP1β* expressor group (Fig. 1A). The OS and DFS time were calculated as the length of time from date of surgical operation to the date of specific breast cancer-related death and relapse/metastasis, respectively. However, expression of other *HP1* subtype mRNAs was not analyzed in this analysis or did not affect the survival in a statistically-significant manner. Furthermore, high *HP1β* expression was associated with poorly differentiated cancer grade (Fig. 1B). High *HP1β* expression group displayed more aggressive types of breast cancers like basal and luminal B type. However, low *HP1β* expressors exhibited more low, moderately, or well-differentiated phenotypes. These suggest that *HP1β* mRNA expression level may be a prognostic marker for survival of breast cancer patients.

### Altered expression of HP1 proteins in breast cancer patients

**Fig 2:**
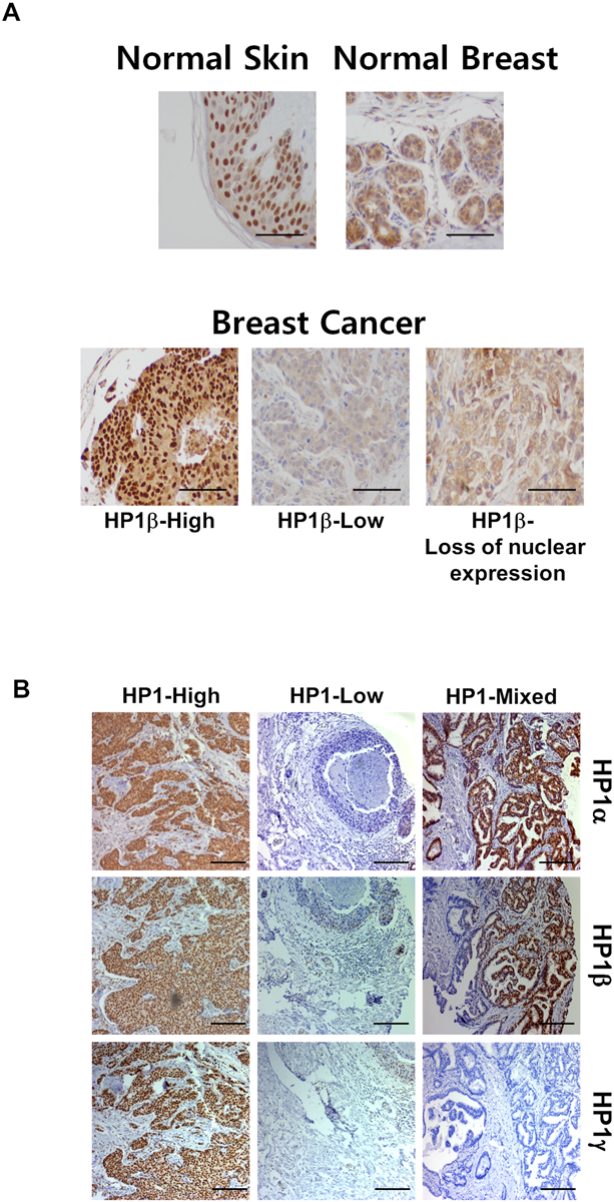
Levels of HP1 protein in breast cancer tissues are heterogeneous. *A*. Normal skin and breast tissues were stained with an anti-HP1β antibody (*upper panel*). Representative examples of breast cancer sample staining by an anti-HP1β antibody (*lower panel*). Depending on expression level and subcellular localization of HP1β, cancer samples were classified to three groups. *B*. Comparison of expression HP1 subtypes in breast cancer samples. Three sets of 190 breast cancer tumors were stained with individual subtypes anti-HP1 antibodies, anti-HP1α, HP1β or HP1γ (Abcam antibodies: ab77256, ab10478, ab10480). IHC staining patterns were compared and IHC staining scores were determined as shown in S1 Fig. Scale bars: 100 μm. HP1-High indicates the group of tumors with abundant expression of all three HP1 subtypes. HP1-Low is the group with no or low expression of all three HP1 subtypes. HP1-Mixed group of cancer samples are high level expression with one or two HP1 subtypes. Representative images are shown.

Since *HP1β* mRNA expression levels were significantly associated with survival of breast cancer patients, we sought to examine the protein expression level of HP1 subtypes in breast cancer samples by IHC staining. First, normal skin and normal breast samples were stained with HP1β specific antibody. IHC of normal skin showed that HP1β staining is nuclear. Similarly, IHC staining showed that HP1β in normal mammary samples also showed that HP1β is primarily nuclear with a weaker expression of HP1β in the cytoplasm (Fig. 2A, *upper panels*). Next, we stained 190 breast cancer samples using an anti-HP1β antibody. Fig. 2A (*lower panels*) shows that HP1β expression patterns in breast cancer samples are diverse and altered in most of cases. Some of the breast cancer samples showed strong nuclear HP1β levels in the nucleus, whereas other samples showed lower or no HP1β signals. There are also samples that manifest clear staining of HP1β only in the cytoplasm, but not in the nucleus (Fig. 2A, *lower panels*). Therefore, HP1β is heterogeneously distributed in breast cancer samples. The breast cancer samples were further analyzed individually and classified into four groups (0, 1, 2, 3) based on the HP1β expression level (S1 Fig.). Accordingly, 34 (18.6%), 39 (21.3%), 58 (31.7%) and 52 (28.4%) samples were designed to group-0, -1, -2 and -3, respectively. We then further divided them into low HP1β expression group (0+1) and high HP1β expression group (2+3). Overall, 60.1% of breast cancer biospecimens exhibited high HP1β (HP1β-high) levels and 39.9% of breast cancer samples showed no or low levels of HP1β (HP1β-low) (Fig. 2).

To compare the expression patterns of other HP1 subtypes, IHC staining was also performed with anti-HP1α or -HP1γ antibody. Fig. 2B (*upper and lower panels*) shows an altered and heterogeneous HP1α and HP1γ staining in breast cancer samples. In parallel, we further classified high and low HP1α and HP1γ level groups based on their respective expression level. 111 (58.4%) and 109 (57.4%) tumors displayed high HP1α and HP1γ expression (S1 and S2 Tables). Taken together, approximately 60% breast cancer patients showed high levels of at least one HP1 subtype. Intriguingly, 82 cases (46.6%) of 176 validated stained samples by all three antibodies showed the same IHC staining scores by antibodies recognizing three individual HP1 subtypes. Fig. 2B shows that HP1-High and HP1-Low group samples were stained (or not) with three individual HP1 subtypes antibodies, respectively. HP1-Mixed is the group of the breast tumors showing strong expression of only one or two HP1 subtype(s). Specifically, 133 breast cancer tumors (75.6%) showed the same groups of high or low HP1 expressors. Only 15% of cancer samples showed a mixed expression pattern of HP1 subtypes (Fig. 2B, HP1-Mixed). Furthermore, analysis of nuclear and cytoplasmic staining patterns showed that HP1 proteins are strongly stained in the cytoplasm of some cancer samples. Especially, 49 cases (27%, out of 176) of breast cancer patients showed strong cytoplasmic HP1α staining. Although a significant portion of cancer tissues showed stronger HP1 cytoplasmic staining in this study (Fig. 2), the potential roles of HP1 mis-localization in breast cancer cells remain unclear. We have compared various cancer markers between HP1-nucleus and HP1-cytoplasm groups (data not shown). However, the correlation of HP1 mis-localization and breast cancer tumorigenesis has yet been established.

### Positive correlation of HP1α, β and γ expression and Ki-67, a cell proliferation marker, in breast cancer

**Fig 3:**
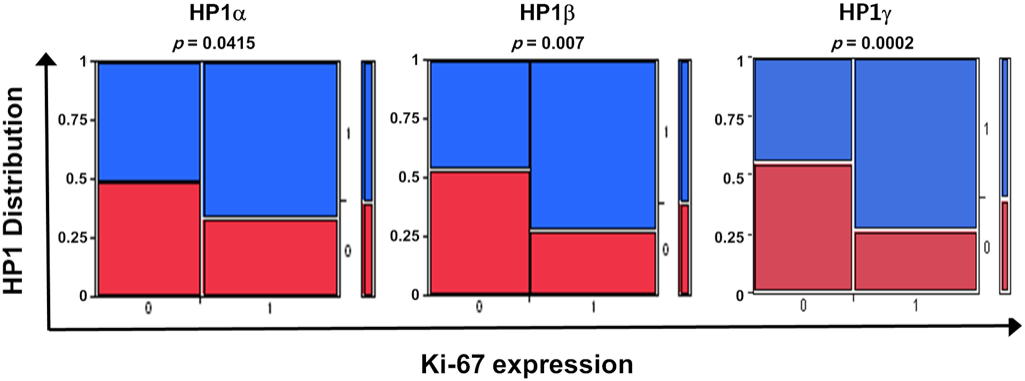
Positive correlation of HP1 and Ki-67 levels in breast cancer. Levels of HP1α, HP1β and HP1γ IHC signal were positively correlated with Ki-67 levels in breast cancer patients. 0 indicates no or low expression and 1 denotes high expression of respective HP1 subtype, as shown in Fig. 2, and Ki-67 level, respectively.

Table 1, S1 and S2 Tables show the clinical and pathological characteristics of 190 breast cancer patients. These include patients’ ages, tumor stages, lymph node infiltration, expression status of ER, PR, p53, Ki-67, HER2. We divided the patient groups into the high HP1 group or the low HP1 group according IHC scoring data. We analyzed the correlation of respective HP1 IHC signals with other breast cancer clinical and pathological markers in Table 1, S1 and S2 Tables. Notably, one common feature of our findings was the positive correlation of Ki-67 with HP1α (*p* = 0.0415), HP1β (*p* = 0.0007) and HP1γ (*p* = 0.0002), respectively (Fig. 3). Our analyses further indicated a significant correlation between HP1α level and several breast cancer markers, such as age, ER status, p53 status and molecular subtypes (S1 Table). HP1γ level was also correlated with p53 status (S2 Table). However, the HP1β signal showed significant correlation especially with Ki-67 (Table 1). Since expressions of all three HP1 subtypes showed a clear correlation with the cell proliferation marker, Ki-67, high HP1 expression probably reflects a group of patients with actively growing breast cancer cells. Conceivably, these analyses suggested the abundance of all three HP1 subtypes could provide useful prognostic information on breast cancer patients.

**Table 1:** Correlation analyses of HP1β expression level with several molecular and pathological cancer markers.

	Characteristics	Total number of patients	HP1β-Low (N = 74)	HP1β-High (N = 113)	*p-value*
**Median age**	Age<49 years	83 (43.9%)	32 (43.2%)	50 (44.2%)	0.3141
	Age>49 years	107 (56.1%)	42 (56.8%)	63 (56.8%)	
**Tumor stages**	T0–T1	58 (31.5%)	24 (36.4%)	32 (28.6%)	0.2818
	T2–T3	123 (68.5%)	42 (63.6%)	80 (71.4%)	
**Lymph node**	N2 negative	98 (54.0%)	45 (60.1%)	56 (49.6%)	0.1301
	N2 positive	92 (46.0%)	29 (39.2%)	57 (50.4%)	
**ER**	ER negative	61 (40.7%)	24 (43.6%)	37 (39.0%)	0.5737
	ER positive	91 (59.3%)	31 (56.4%)	58 (61.0%)	
**PR**	PR negative	71 (47.4%)	22 (40.7%)	50 (51.0%)	0.2234
	PR positive	83 (52.6%)	32 (59.3%)	48 (49.0%)	
**p53**	p53 negative	92 (57.6%)	40 (66.7%)	51 (52.0%)	0.0693
	p53 positive	67 (42.4%)	20 (33.3%)	47 (48.0%)	
**Ki-67**	Ki-67 negative	72 (44.5%)	39 (60.9%)	34 (34.0%)	**0.0007**
	Ki-67 positive	94 (55.5%)	25 (39.1%)	66 (66.0%)	
**HER2**	HER2 negative	129 (82.2%)	45 (83.3%)	80 (81.6%)	0.069
	HER2 positive	27 (17.8%)	9 (16.7%)	18 (18.4%)	
**Molecular type**	Luminal A	50 (35.5%)	23 (47.9%)	26 (28.9%)	0.1179
	Luminal B	52 (37.0%)	15 (31.3%)	36 (40.0%)	
	TNBC	26 (18.8%)	8 (16.7%)	18 (20.0%)	
	HER2+	12 (8.7%)	2 (4.2%)	10 (11.1%)	

### HP1β depleted breast cancer cells are hypersensitive to PARP inhibitor

**Fig 4:**
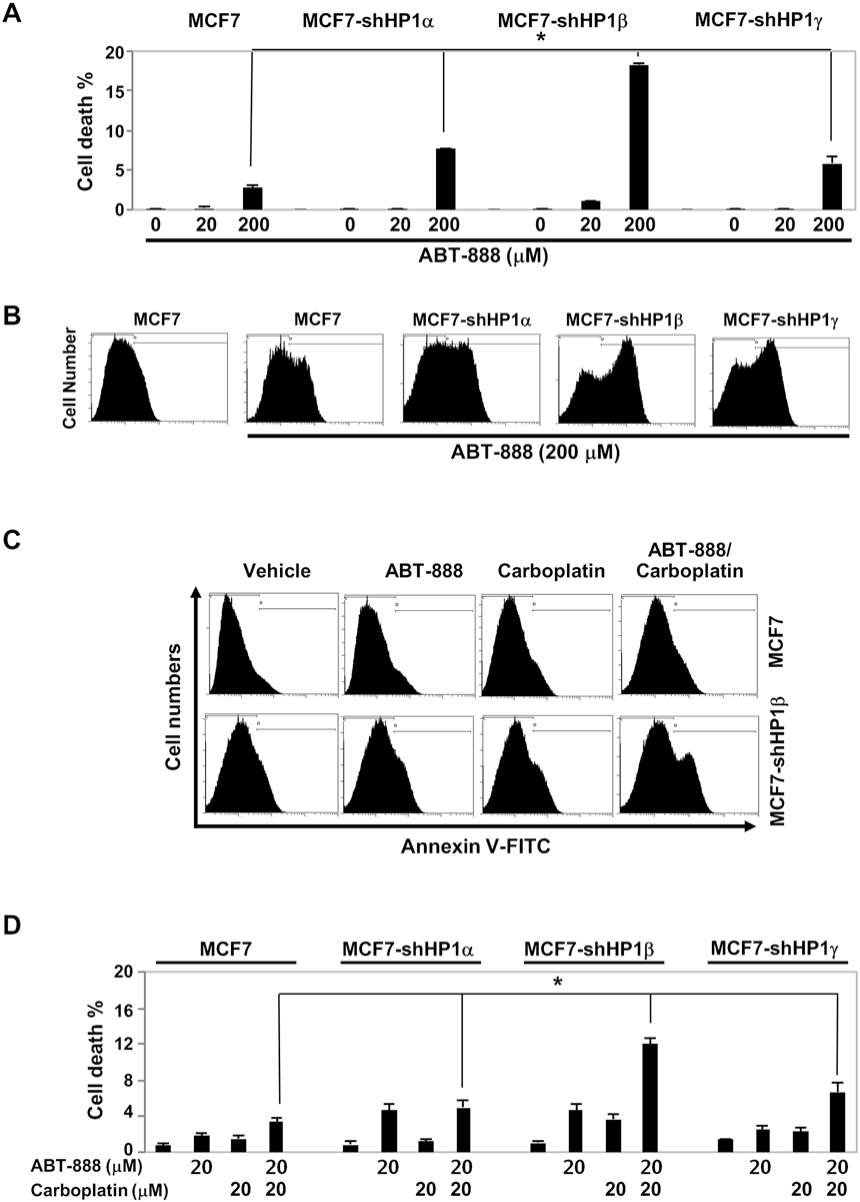
PARP inhibitor, ABT-888, induces apoptosis in HP1-depleted breast cancer cells. ABT-888 (20 μM or 200 μM) was used to treat MCF7 and individually HP1-depleted MCF7 cells for 72 hours. Cells were stained with Annexin V and propidium iodide staining and cell population was analyzed by flow cytometry. *A*. Cell death percentage on Y-axis is for the double stained population using Annexin V (FITC-conjugated) and propidium iodide. *B*. Annexin V stained cell population was analyzed by flow cytometry. Y axis: cell numbers, X axis: FL1 values (Annexin V). *C*. HP1β-depleted MCF7 breast cancer cells are more prone to apoptosis by ABT-888 and carboplatin. Apoptosis of MCF7 and individually HP1-depleted MCF7 cells was determined after treatment of ABT-888 (20 μM), carboplatin (20 μM) or ABT-888/carboplatin combination (20 μM/20 μM) for 72 hours. A representative Annexin V staining of MCF7 and HP1β depleted MCF7 cells. *D*. Percentage of apoptotic MCF7 and HP1-depleted MCF7 cells were determined by double staining with Annexin V and propidium iodide followed by analysis using flow cytometry. _*_: *p* < 0.05 from analysis of three independent assays, Student's t-test.

Previously, we reported that HP1 family is required for DNA damage response primarily through the regulation of BRCA1 function [17]. HP1-depleted cells showed defective BRCA1 foci formation, homologous recombination DNA repair and G2/M cell cycle checkpoint control in response to irradiation. As this study showed that significant populations of breast cancer patients have low or no expression of at least one HP1 subtype (Fig. 2), we tested the effect of individual HP1 on PARP inhibitor therapy. To achieve this goal, MCF7 cells and individually HP1-depleted MCF7 cells (S2 Fig.) were treated with ABT-888 (Veliparib), which is one of the PARP inhibitors currently undergoing clinical evaluation [25]. MCF7 breast cancer cells were chosen for our experimental paradigm because MCF7 harbors wild-type tumor suppressor BRCA1 in addition to wild type p53, ER, and PR [26]. MCF7 cells and individually HP1-depleted MCF7 cells were treated with vehicle or ABT-888 (20 or 200 μM) for 72 hours. The cells were collected and stained with Annexin V and propidium iodide (PI) and analyzed by flow cytometry. MCF7 cells with wild type BRCA1 were relatively resistant to PARP inhibitor treatment (Fig. 4A). However, treatment of ABT-888 (20 μM) induced high level of apoptosis in HP1β-depleted MCF7 cells. Although treatment ABT-888 (200 μM) barely increased the Annexin V-positive MCF7 population, it markedly increased Annexin V-positive, presumably apoptotic, HP1α-, β- or γ-knockdown MCF7 cells (Fig. 4B). Notably, the cell death (double stained population by PI and Annexin V) in HP1β-depleted cells was 10 times higher than that of MCF7 cells (Fig. 4A). HP1α- or γ- depleted MCF7 cells were also hypersensitive to ABT-888. This suggests that PARP inhibitor ABT-888 can effectively target HP1-deficient, especially HP1β-deficient, breast cancer cells.

We then examined the combination effects of ABT-888 and carboplatin on apoptosis of MCF7 cells and individually HP1-depleted MCF7 cells. Carboplatin is an alkylating agent that exhibits a cytotoxic effect on cancer cells by binding to DNA and forming interstrand crosslinks that block DNA replication. Previously, the synthetic lethality of ABT-888 and carboplatin in breast cancer cells with respect to BRCA status was reported *in vitro* and *in vivo* [27]. To test the effect of HP1 status on the synthetic lethality of these two drugs, MCF7 cells and individually HP1-depleted MCF7 cells were treated with a combination of ABT-888 (20 μM) and carboplatin (20 μM). As shown in Fig. 4C, neither ABT-888 alone, carboplatin alone nor combination had marked effect on rendering Annexin V-positive in MCF7 cells. However, same amounts of ABT-888 or carboplatin induced cell death of HP1β-depleted MCF7 cells (Fig. 4D). Notably, combination of ABT-888 and carboplatin resulted in marked cytotoxic effects in HP1β-depleted MCF7 cells. These results showed that PARP inhibitors and/or carboplatin can be an effective therapy regimen for patients with breast cancer of no or low HP1β expressors. Conceivably, HP1α or HP1γ deficiency in tumor tissues can be translated as a predictive marker for breast cancer PARP inhibitor therapy. While HP1α and HP1γ compromised MCF7 cells showed 2~3 fold higher sensitivity to PARP inhibitor treatment, HP1β deficient cells were much more sensitive to PARP inhibitor (Fig. 4). In other words, HP1 levels, especially HP1β deficiency, could be a useful predicative marker for BRCAness for the effective use of PARP therapy.

## Discussion

### HP1 is a potential prognostic marker for breast cancer

Identification of novel biomarkers for breast cancer is crucial for predicting cancer prognosis and therapeutic outcomes [28]. The diverse genetic variations and mutations found in breast cancers make it difficult to classify those tumors into groups to improve therapeutic guidance. Therefore, identification of additional molecular signatures of breast cancers will provide a better basis for targeted therapy and personalized medicine. Herein, results presented in this study suggest that high levels of HP1β are a poor prognostic marker for breast cancer outcome (Fig. 1). Moreover, high HP1 expressors may indicate a group of patients harboring actively growing breast cancer cells, since all HP1α, β and γ expression correlated with Ki-67, a surrogate marker for cell proliferation (Fig. 3). Lastly, lack-of-HP1β-expression could serve as a predictive marker to define a breast cancer therapeutic option (Fig. 4).

Previously, several groups have shown that HP1 subtype levels were either decreased or increased in several cancers and tissues [29]. However, the results from analyzing the levels of HP1α in breast cancers, in general, are still controversial. For example, Kirschmann et al. showed that expression level of HP1α was decreased in metastatic and aggressive breast cancer cells [30]. In contrast, another group demonstrated HP1α expression is upregulated in breast cancer tumor samples [31]. In this study, we analyzed the expression levels of all three types of HP1 in breast cancer biospecimens by a combined data mining of published microarray data and IHC study. Here we show that the mRNA and protein expression levels of HP1 are frequently altered and diverse among breast cancer biospecimens. *HP1β* mRNA levels are inversely correlated with survival (OS and DFS) of breast cancer patients (Fig. 1). HP1α protein levels showed a correlation with several cancer markers including age, p53 status, ER status and Ki-67 (Table 1 and Fig. 3). However, expressions of all three subtypes of HP1 are frequently regulated in similar manner in cancer cells (Fig. 2). Our results reveal that all three HP1 subtypes are potentially useful markers for breast cancer prognosis. Notably, expression levels of HP1 showed strong correlation with Ki-67 level in breast cancer samples (Fig. 3). Ki-67 is used as an indicator to further classify triple negative breast cancers [32]. Analysis of HP1 expression in cancer patients may also be useful for further analyzing breast cancer molecular subtypes. Previously other groups showed that breast cancer cells with high HP1α are more prone to cell cycle progression [31]. This is consistent with our finding showing a positive correlation of HP1α and cell proliferation marker Ki-67. Furthermore, our study shows that there is a strong correlation of Ki-67 expression with other HP1 subtypes. Further investigation of the relation between expression of HP1 subtypes and Ki-67 in other cancers including prostate cancer could also be worthwhile [33]. Our results together with other reports suggest the potential significance of HP1 in breast cancer prognosis and thus this warrants additional studies.

### Potential roles of HP1 in carcinogenesis

**Fig 5:**
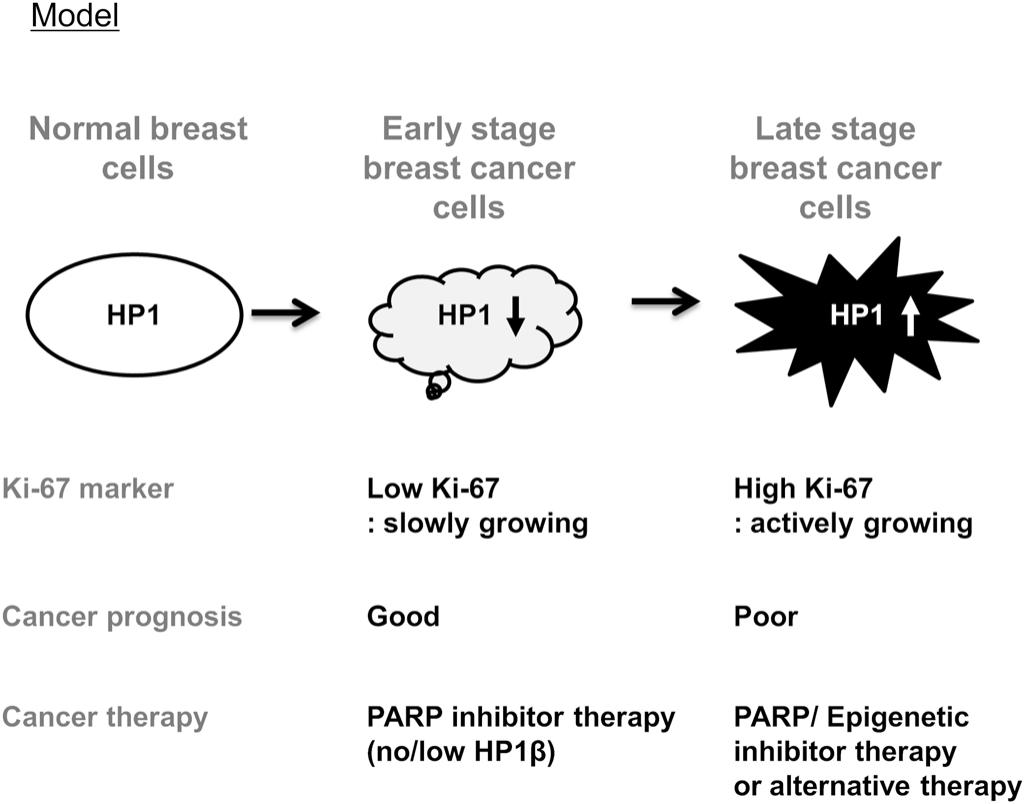
HP1β is a biomarker for breast cancer prognosis and PARP inhibitor therapy. Respective HP1 expression level is frequently altered in breast cancer cells, suggesting the diverse role of each HP1 subtype in breast cancers. This model shows that the expression level of HP1 subtype in breast cancer cells may be either decreased or increased according to cancer stage, grade, cancer cell proliferation (Ki-67) and aggressiveness. PARP inhibitor therapy may be an effective therapy for patients with no/low HP1β expression. Combination therapy with epigenetic drugs (including H3K9 methylation inhibitors) or alternative therapy is necessary for patients with breast cancers of high HP1 abundance.

While the complicated HP1 levels and pattern in breast cancer biospecimens could also reflect the heterogeneity of cancer cells in human breast tumors [6, 34], it is intriguing that expression levels of three HP1 subtypes were comparably regulated in some breast cancer cells. These altered and heterogeneous staining patterns also implicate that HP1 family plays diverse roles in breast cancers. As HP1 subtypes elicit multiple functions in cells, we surmise that the expression levels and subcellular location of HP1 are dynamically regulated during tumorigenesis (Fig. 5). Previously we showed that HP1 is required for homologous recombination repair and cell cycle control through the regulation of BRCA1 [17]. HP1 is also involved in the other cellular functions, such as transcription and cell proliferation. Thereby, we speculate that the lack-of-HP1-expression in some breast cancer tumors can deregulate their BRCA1 functions in homologous recombination repair and cell cycle checkpoint control. Conceivably, genomic mutations could accumulate in cancer cells with low HP1 levels. This could explain why some cancer patients exhibited lack-of-HP1-expression phenotypes in cancer cells.

However, it is not clear how high HP1 expression contributes to tumorigenesis. High levels of HP1 may deregulate the expression of genes involved in tumorigenesis, thereby promoting the growth and proliferation of cancer cells. This possibility is supported by the observation showing a significant correlation of HP1 expression with Ki-67 level (Fig. 3, Table 1, S1, S2 Tables). Ki-67 is a nuclear protein that correlates with cell cycle progression through S-phase [35]. It is widely held that Ki-67 exists at low levels in normal and resting (G0 phase) cells. This is why Ki-67 is considered to be a surrogate marker for cell proliferation and also a poor prognostic marker for several cancers, including breast cancer [36–40]. More recently, HP1γ and Ki-67 levels in prostate cancer cases were correlated [33]. We propose that high HP1 expression can be used as a breast cancer marker like Ki-67, indicating actively growing cancer cells, as does Ki-67. This possibility is supported by several reports demonstrating that HP1 forms a complex with Ki-67 through the C-terminal domain of Ki-67 [41, 42]. It is likely that the HP1 and Ki-67 complex is regulated simultaneously and plays critical roles in tumorigenesis.

### HP1β is a potential predictive marker for PARP inhibitor therapy

Importantly, our results shown in Fig. 4 clearly suggest that ABT-888, a PARP inhibitor, is more effective in removing low HP1-expressing, especially low HP1β-expressing, breast cancer cells by apoptosis. Conceivably, we propose that PARP inhibitor therapy could be an effective therapy not only for patients with BRCA1/2 mutations but also for patients with no/low HP1β expressions. However, it is not clear what is the therapeutic recommendation for breast cancer groups with high HP1 expression. It is possible that HP1-high patient group could benefit from either combination therapy of PARP inhibitor/epigenetic drugs or alternative therapy (Fig. 5). Alternative therapeutic strategies could be a better option for breast cancer patients with high HP1 expression. Since HP1 plays critical roles in heterochromatin maintenance, we further speculate that the effects of high HP1 abundance in cancer cells to be overcome by drugs affecting chromatin structure including HDAC (histone deacetylase) inhibitors or H3K9 (histone H2 Lysine 9) methylation inhibitors.

One of the caveats of PARP inhibitor therapy is the selectivity of the drug in killing particular cancer cells [12, 13]. PARP inhibitor can selectively kill BRCA1-deficient and HR-repair deficient cancer cells [10]. PARP therapy could be an important therapeutic option for breast cancer, ovarian cancer and other cancers and clinical trials of PARP inhibitor are currently in progress [14–16]. One of the limitations of PARP therapy is that there are limited numbers of cancer patients with BRCA1 or BRCA2 mutation. If this experimental finding holds in pre-clinical or clinical studies, many more breast cancer patients could benefit from PARP inhibitor therapy, because HR repair is deficient in many cancers without BRCA1 or BRCA2 mutations. This so-called BRCAness phenomenon was reported previously in breast, ovarian and other cancer cases [9, 15, 43–45]. Impaired homologous recombination repair can be caused by epigenetic DNA methylation of promoters or by mutations of DNA damage response regulators [9]. Since we showed that HP1-deficiency impaired homologous recombination repair and rendered BRCAness phenotype in breast cancer cells [17], we confirmed the cytotoxicity of PARP inhibitor for HP1-deficient breast cancer cells (Fig. 4). To the best of our knowledge, there is no standard assay to detect BRCAness [15]. This study indicates that analysis of HP1β expression level can be an informative predictive biomarker for BRCAness and for inducing synthetic lethality of breast cancer cells by PARP inhibition. Thus, analysis of HP1β level in breast tumors not only provides a breast cancer prognosis biomarker but also a predictor for PARP inhibitor therapy.

## Supporting Information

S1 Fig IHC scoring standard for breast cancer samples. Breast cancer samples were stained with an anti-HP1β antibody. IHC scores of each breast cancer samples were scored according to the intensity of staining. This standard staining 0 shows no staining by HP1β. Standard 3 shows the strong staining. IHC scoring was performed according to this staining standard.
(TIF)

S2 Fig Western blot analysis for MCF7 and HP1-depleted MCF7 cells by HP1-specific antibodies. MCF7 cells were infected with lentiviral vectors harboring shRNAs for each HP1 subtypes [17]. Knockdown efficiency of HP1 in MCF7 cells are analyzed by Western blot with specific HP1 antibodies.
(TIF)

S1 Table Contingency analysis of HP1α in breast cancer samples. (DOCX)

S2 Table Contingency analysis of HP1γ in breast cancer samples. (DOCX)
