# Correction: HP1β Is a Biomarker for Breast Cancer Prognosis and PARP Inhibitor Therapy

**DOI:** 10.1371/journal.pone.0124853

**Published:** 2015-04-13

**Authors:** Young-Ho Lee, Xiyong Liu, Fuming Qiu, Timothy R. O’Connor, Yun Yen, David K. Ann

There are errors in the Funding section. The correct funding information is as follows: This work was supported by National Institute of Health Research Grants R01DE10742 and R01DE14183, and The Mary Kay Foundation Research Grant number 005–13 (to DKA). The funders had no role in study design, data collection and analysis, decision to publish, or preparation of the manuscript.
