# Supplementary figures and images for: HP1β Is a Biomarker for Breast Cancer Prognosis and PARP Inhibitor Therapy
Source: PLoS One. 2015 Mar 13;10(3):e0121207. doi: 10.1371/journal.pone.0121207 (PMC4358987; doi:10.1371/journal.pone.0121207)

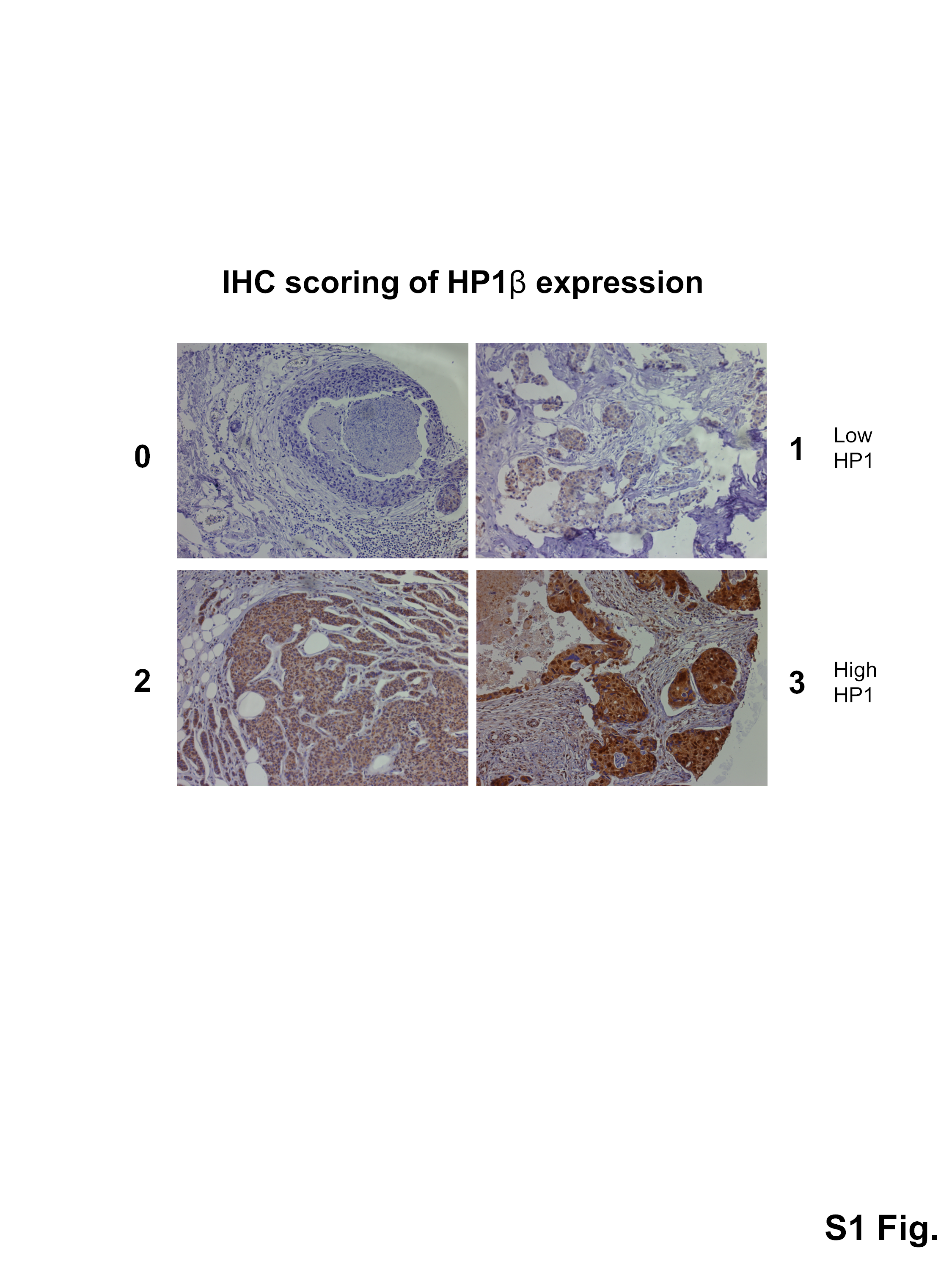

Supplement: S1 Fig — Breast cancer samples were stained with an anti-HP1β antibody. IHC scores of each breast cancer samples were scored according to the intensity of staining. This standard staining 0 shows no staining by HP1β. Standard 3 shows the strong staining. IHC scoring was performed according to this staining standard. (TIF) [file pone.0121207.s001.TIF]

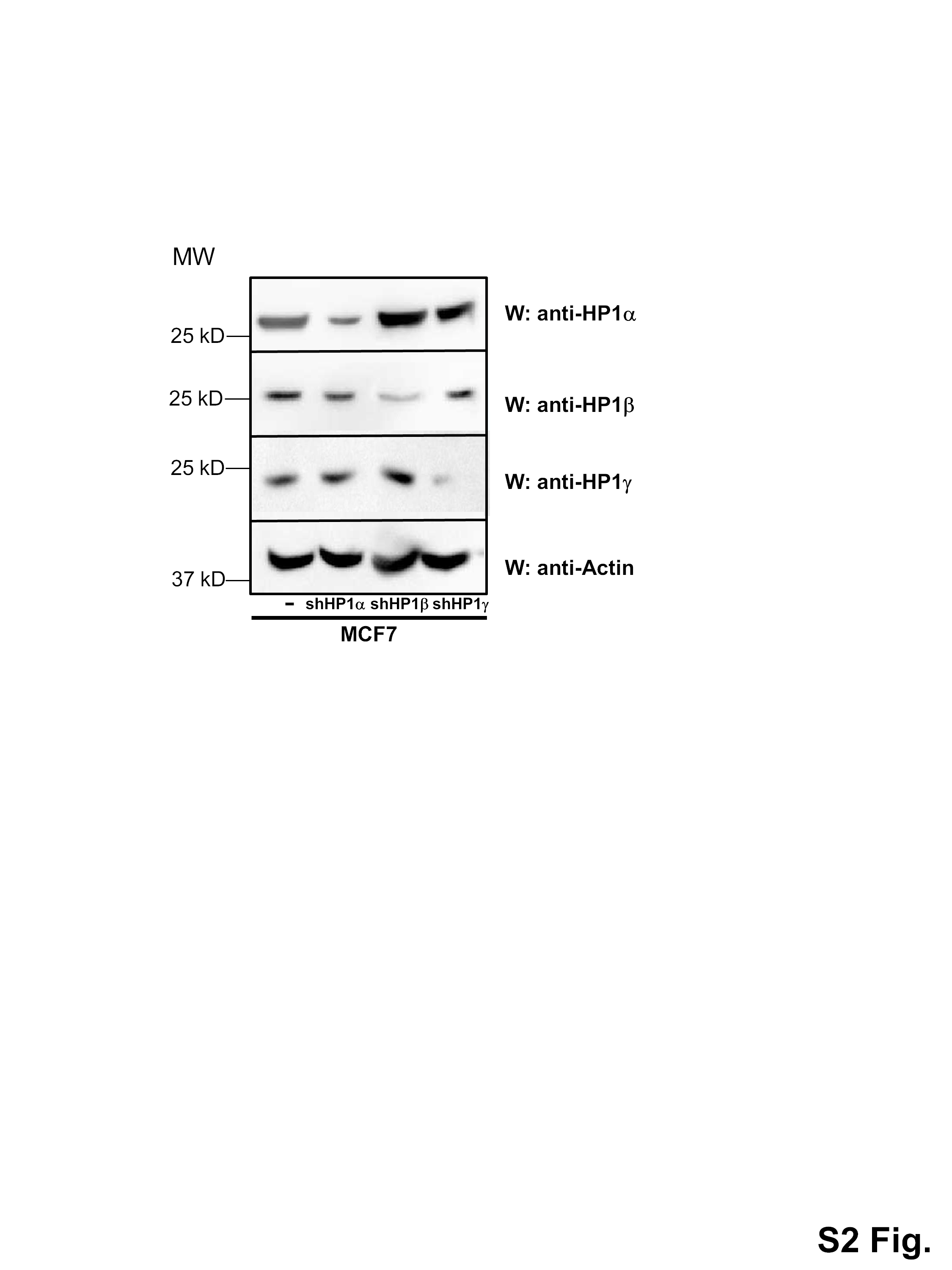

Supplement: S2 Fig — MCF7 cells were infected with lentiviral vectors harboring shRNAs for each HP1 subtypes [17]. Knockdown efficiency of HP1 in MCF7 cells are analyzed by Western blot with specific HP1 antibodies. (TIF) [file pone.0121207.s002.TIF]
