# Supplementary material for: HP1β Is a Biomarker for Breast Cancer Prognosis and PARP Inhibitor Therapy
Source: PLoS One. 2015 Mar 13;10(3):e0121207. doi: 10.1371/journal.pone.0121207 (PMC4358987; doi:10.1371/journal.pone.0121207)
Supplement: S1 Table — (DOCX) [file pone.0121207.s003.docx]

**S1 Table. Correlation analyses of HP1α expression level with several molecular and pathological cancer markers.**

|  | **Characteristics** | **Total number of patients** | **HP1α-Low**  (N = 79) | **HP1α-High**  (N = 111) | ***p-value*** |
| --- | --- | --- | --- | --- | --- |
| **Median age** | Age<49 years | 83 (43.9 %) | 28 (35.4 %) | 55 (44.2 %) | **0.0164** |
|  | Age>49 years | 107 (56.1 %) | 51 (64.6 %) | 56 (56.8 %) |  |
| **Tumor stages** | T0-T1 | 58 (32.0 %) | 26 (35.1 %) | 32 (29.9 %) | 0.4596 |
|  | T2-T3 | 123 (68.0 %) | 48 (64.9 %) | 75 (70.1 %) |  |
| **Lymph node** | N2 negative | 98 (51.6 %) | 43 (54.4 %) | 55 (49.6 %) | 0.5068 |
|  | N2 positive | 92 (48.4 %) | 36 (45.6 %) | 56 (50.4 %) |  |
| **ER** | ER negative | 61 (40.1 %) | 33 (54.1 %) | 28 (30.8 %) | **0.004** |
|  | ER positive | 91 (59.9 %) | 28 (45.9 %) | 63 (69.2 %) |  |
| **PR** | PR negative | 71 (46.1 %) | 26 (44.1 %) | 45 (47.4 %) | 0.6894 |
|  | PR positive | 83 (53.9 %) | 33 (55.9 %) | 50 (52.6 %) |  |
| **p53** | p53 negative | 92 (57.9 %) | 43 (68.3 %) | 49 (51.0 %) | **0.0304** |
|  | p53 positive | 67 (42.1 %) | 20 (31.8 %) | 47 (49.0 %) |  |
| **Ki-67** | Ki-67 negative | 72 (43.4 %) | 35 (53.0 %) | 37 (37.0 %) | **0.0415** |
|  | Ki-67 positive | 94 (56.6 %) | 31 (47.0 %) | 63 (63.0 %) |  |
| **HER2** | HER2 negative | 129 (82.7 %) | 50 (86.2 %) | 79 (80.6 %) | 0.3657 |
|  | HER2 positive | 27 (17.3 %) | 8 (13.8 %) | 19 (19.4 %) |  |
| **Molecular type** | Luminal A | 50 (35.7 %) | 22 (40.7 %) | 28 (32.6 %) | **0.0245** |
|  | Luminal B | 52 (37.1 %) | 15 (20.8 %) | 37 (43.0 %) |  |
|  | TNBC | 26 (18.6 %) | 15 (27.8 %) | 11 (12.8 %) |  |
|  | HER2+ | 12 (8.6 %) | 2 (3.7 %) | 10 (11.6 %) |  |
