# Supplementary material for: HP1β Is a Biomarker for Breast Cancer Prognosis and PARP Inhibitor Therapy
Source: PLoS One. 2015 Mar 13;10(3):e0121207. doi: 10.1371/journal.pone.0121207 (PMC4358987; doi:10.1371/journal.pone.0121207)
Supplement: S2 Table — (DOCX) [file pone.0121207.s004.docx]

**S2 Table. Correlation analyses of HP1γ expression level with several molecular and pathological cancer markers.**

|  | **Characteristics** | **Total number of patients** | **HP1γ-Low**  (N = 81) | **HP1γ-High**  (N = 109) | ***p-value*** |
| --- | --- | --- | --- | --- | --- |
| **Median age** | Age<49 years | 86 (45.3 %) | 34 (42.0 %) | 52 (47.7 %) | 0.3998 |
|  | Age>49 years | 104 (54.7 %) | 47 (58.0 %) | 57 (52.3 %) |  |
| **Tumor stages** | T0-T1 | 58 (31.9 %) | 27 (33.3 %) | 31 (28.4 %) | 0.943 |
|  | T2-T3 | 124 (68.1 %) | 54 (66.7 %) | 78 (71.6 %) |  |
| **Lymph node** | N2 negative | 99 (52.1 %) | 43 (53.1 %) | 56 (51.4 %) | 0.054 |
|  | N2 positive | 91 (47.9 %) | 38 (46.9 %) | 53 (48.6 %) |  |
| **ER** | ER negative | 59 (39.6 %) | 25 (44.6 %) | 34 (36.6 %) | 0.3295 |
|  | ER positive | 90 (60.4 %) | 31 (55.4 %) | 59 (63.4 %) |  |
| **PR** | PR negative | 73 (47.4 %) | 24 (42.1 %) | 49 (50.5 %) | 0.3122 |
|  | PR positive | 81 (52.6 %) | 33 (57.9 %) | 48 (49.5 %) |  |
| **p53** | p53 negative | 91 (58.0 %) | 42 (70.0 %) | 49 (50.5 %) | **0.0153** |
|  | p53 positive | 66 (42.0 %) | 18 (30.0 %) | 48 (49.5 %) |  |
| **Ki-67** | Ki-67 negative | 74 (44.6 %) | 41 (65.1 %) | 33 (33.0 %) | **0.0002** |
|  | Ki-67 positive | 92 (55.4 %) | 25 (37.9 %) | 67 (67.0 %) |  |
| **HER2** | HER2 negative | 127 (81.4 %) | 49 (83.1 %) | 78 (80.4 %) | 0.170 |
|  | HER2 positive | 29 (18.6 %) | 10 (16.9 %) | 19 (19.6 %) |  |
| **Molecular type** | Luminal A | 49 (35.5 %) | 24 (48.0 %) | 25 (28.4 %) | 0.0603 |
|  | Luminal B | 51 (37.0 %) | 14 (28.0 %) | 37 (42.1 %) |  |
|  | TNBC | 26 (18.8 %) | 10 (20.0 %) | 16 (18.2 %) |  |
|  | HER2+ | 12 (8.7 %) | 2 (4.0 %) | 10 (11.4 %) |  |
